# Deletion of Btg1 Induces Prmt1-Dependent Apoptosis and Increased Stemness in Shh-Type Medulloblastoma Cells Without Affecting Tumor Frequency

**DOI:** 10.3389/fonc.2020.00226

**Published:** 2020-03-13

**Authors:** Manuela Ceccarelli, Giorgio D'Andrea, Laura Micheli, Felice Tirone

**Affiliations:** Institute of Biochemistry and Cell Biology, National Research Council (IBBC-CNR), Rome, Italy

**Keywords:** cerebellum neurogenesis, medulloblastoma, neoplastic granule cell precursors, apoptosis, proliferation, protein arginine methyltransferase 1 (Prmt1), B-cell translocation gene 1 (Btg1), Sonic hedgehog (Shh)

## Abstract

About 30% of medulloblastomas (MBs), a tumor of the cerebellum, arise from cerebellar granule cell precursors (GCPs) undergoing transformation following activation of the Sonic hedgehog (Shh) pathway. To study this process, we generated a new MB model by crossing *Patched1* heterozygous (*Ptch1*^+/−^) mice, which develop spontaneous Shh-type MBs, with mice lacking B-cell translocation gene 1 (*Btg1*), a regulator of cerebellar development. In MBs developing in *Ptch1*^+/−^ mice, deletion of *Btg1* does not alter tumor and lesion frequencies, nor affect the proliferation of neoplastic precursor cells. However, in both tumors and lesions arising in *Ptch1*^+/−^ mice, ablation of *Btg1* increases by about 25% the apoptotic neoplastic precursor cells, as judged by positivity to activated caspase-3. Moreover, although *Btg1* ablation in early postnatal GCPs, developing in the external granule cell layer, leads to a significant increase of proliferation, and decrease of differentiation, relative to wild-type, no synergy occurs with the *Ptch1*^+/−^ mutation. However, *Btg1* deletion greatly increases apoptosis in postnatal GCPs, with strong synergy between *Btg1*-null and *Ptch1*^+/−^ mutations. That pronounced increase of apoptosis observed in *Ptch1*^+/−^*/Btg1* knockout young or neoplastic GCPs may be responsible for the lack of effect of *Btg1* ablation on tumorigenesis. This increased apoptosis may be a consequence of increased expression of protein arginine methyltransferase 1 (Prmt1) protein that we observe in *Btg1* knockout/*Ptch1*^+/−^ MBs. In fact, apoptotic genes, such as *BAD*, are targets of Prmt1. Moreover, in *Btg1*-null MBs, we observed a two-fold increase of cells positive to CD15, which labels tumor stem cells, raising the possibility of activation of quiescent tumor cells, known for their role in long-term resistance to treatment and relapses. Thus, *Btg1* appears to play a role in cerebellar tumorigenesis by regulating the balance between apoptosis and proliferation during MB development, also influencing the number of tumor stem cells.

## Introduction

B-cell translocation gene 1 (*Btg1*) belongs to the *Btg*/*Tob* gene family, comprising six genes endowed with antiproliferative properties; it shares about 65% homology with the founding member of this gene family *PC3*^*TIS*21/*BTG*2^ (1, 2).

*Btg1* encodes a regulatory cofactor, which interacts with various cellular targets and modulates their activity. These molecular partners include the protein arginine methyltransferase 1 (PRMT1) (3), the transcription factor HoxB9 (4), several nuclear receptors and transcription factors involved in the myoblast differentiation (5), and the Ccr4-associated factor 1 (CAF1), subunit of the CCR4–NOT complex (6). The Btg1 protein functions as a cell cycle inhibitor, with a range of effects on various cellular processes, such as proliferation, differentiation, and apoptosis, in multiple cell types. Btg1 negatively regulates cell proliferation in several cell types, such as NIH3T3 murine fibroblasts (1), macrophages (7), erythroid colonies (8), microglia (9), myoblasts (10), and brain cells (see below) (11, 12). Such a growth arrest may be accompanied by increased apoptotic frequency, as seen in NIH3T3 cells (13) and in microglia (9), or, more frequently, by stimulation of terminal differentiation, as in myoblasts (10) and in erythroid progenitors (8).

Genetic aberrations in *Btg1* are common in B-cell malignancies (14), but this gene is also implicated in different types of solid tumors. In fact, deregulated expression of *Btg1* is involved in gastric (15), kidney (16), liver (17), thyroid (18), nasopharyngeal (19), ovarian (20), breast (21, 22), and non-small-cell lung cancers (23), and is frequently associated with disease severity. Notably, although *Btg1* is expressed in the developing and adult brain (11, 24–26), its involvement in brain tumors has been investigated only in gliomas (27, 28).

In the adult neurogenic niches, i.e., the dentate gyrus of the hippocampus and the subventricular zone, Btg1 is required for the physiological maintenance of the stem/progenitor cells quiescence and self-renewal (11, 29). In the cerebellum, Btg1 negatively controls the proliferation of the cerebellar granule cell precursors (GCPs), playing a critical role for cerebellar development and function (12). In this report, we tested whether Btg1 is also involved in tumorigenesis of the cerebellum.

During postnatal cerebellar morphogenesis, the GCPs intensely proliferate at the surface of the cerebellar primordia to form the external granule layer (EGL): this transient amplification—which in the mouse continues until the second week after birth—is triggered by the diffusible factor Sonic hedgehog (Shh), secreted by the neighboring Purkinje neurons (30–32). Once post-mitotic, the GCPs migrate inward to the molecular and internal granule layers (ML and IGL, respectively) and differentiate in mature granule neurons (33, 34). Thus, a misregulation in these processes may prolong and/or affect the mitotic activity of GCPs at the cerebellar surface, and promote cellular transformation (35), with consequent formation of medulloblastoma (MB). MB is the most common childhood brain tumor and arises in about 30% of cases from GCPs (36–39).

A recent study by us demonstrated that, during the development of the cerebellum, Btg1 is required for the negative control of GCP proliferation, with secondary effects on cellular differentiation, survival, and migration—the latter being essentially dependent on the family-related gene *PC3*^*TIS*21/*BTG*2^ (12). The cell cycle control by Btg1 takes place at the G1/S transition, by selective regulation of *cyclin D1*. We found that in mice, *Btg1* ablation causes hyperproliferation of GCPs and increase of EGL thickness, which remains hyperplastic for a longer period, instead of being progressively reduced. Moreover, we observed *in vitro* that the overexpression of *Btg1* is able to inhibit the proliferation of the MB cell line DAOY (12), thus highlighting *Btg1* as an MB suppressor and as an obvious candidate for MB pathogenesis.

Furthermore, the family-related gene *PC3*^*TIS*21/*BTG*2^ is an MB suppressor, as its overexpression inhibits the proliferation and facilitates the differentiation of neoplastic GCPs (40, 41). In fact, we found that in a mouse model of spontaneous MB, *PC3*^*TIS*21/*BTG*2^ deletion causes a great increase of tumor frequency due to a deficit of GCP migration (42, 43).

Thus, in this study, we tested if the loss of the Btg1-dependent control of GCP proliferation is able to increase the spontaneous tumor incidence in an Shh-MB model, crossing *Btg1* knockout mice to *Patched1* heterozygous (*Ptch1*^+/−^) mice.

Surprisingly, we found that genetic ablation of *Btg1* in *Ptch1*^+/−^ mice does not result in an enhancement of the MB frequency, neither in the preneoplastic stages nor in the established tumors, as it does not affect the proliferation of neoplastic GCPs but significantly increases their apoptosis. This induction of apoptosis in *Btg1*-null GCPs, also observed during cerebellar development, could be due to an increase in Prmt1-mediated arginine methylation of Bcl-2 antagonist of cell death (BAD), which is known to inhibit Akt-dependent BAD phosphorylation, thus promoting its mitochondrial localization and leading to an increase of apoptosis (44). Interestingly, in MBs, the augmented cell death could make a selection of quiescent CD15^+^ tumor stem cells, which are increased in MBs lacking the *Btg1* gene.

## Materials and Methods

### Mouse Lines

*Ptch1*^+/−^ mice were obtained in CD1 background through ablation of exons 6 and 7 (45). The *Btg1* knockout mice were generated in the C57BL/6 strain by replacing the exon I of the gene with the neomycin resistance cassette, as previously described (11).

The crossing of *Ptch1*^+/−^ with *Btg1*^−/−^ mice generated *Ptch1/Btg1* double-mutant mice, which were interbred for at least six generations to obtain an isogenic progeny of the different genotypes under study. To identify the preneoplastic lesions, *Ptch1*/*Btg1* double-mutant mice were crossed with *Math1-GFP* transgenic mice, which express the green fluorescent protein (GFP) driven by the *Math1* enhancer in proliferating GCPs (46, 47), kindly provided by Jane Johnson (UT Southwestern Medical School, Dallas, TX, USA); the *Ptch1/Btg1/Math1-GFP* mice were then interbred four or more times before starting any analysis.

The *Ptch1*/*Btg1* pups were routinely genotyped by PCR analysis using genomic DNA extracted from tail tips, as described (12, 40, 42, 45); the mice which carry the transgene *Math1-GFP* have been previously identified using a “GFP flashlight” (Nightsea) that made the GFP^+^ pups glow.

All animal experiments were performed with mice of both sexes, in accordance with the current European Ethical Committee guidelines (directive 2010/63/EU) and with the protocol approved by the Italian Ministry of Health (authorization 193/2015-PR).

### Treatment of Mice and Sample Preparation

Mice were observed daily for MB formation for 12 months after birth. On the appearance of tumor symptoms, such as head doming, ruffling of fur, hunched posture, impaired balance, severe weight loss, posterior paralysis, or lethargy, they were euthanized under anesthesia and autopsied. MBs were either snap frozen in liquid nitrogen for analyses of mRNAs and proteins or fixed in 4% paraformaldehyde (PFA) in phosphate buffered saline (PBS) by overnight immersion.

For quantification of the lesions, asymptomatic *Ptch1*^+/−^*/Btg1*^+/+^*/Math1-GFP*^+^ and *Ptch1*^+/−^*/Btg1*^−/−^*/Math1-GFP*^+^ mice were killed at different times (2 or 6 weeks of age), and their cerebella were fixed by immersion overnight in 4% PFA in PBS. For the EGL studies, cerebella of postnatal day 7 (P7) mice were dissected out and kept overnight in 4% PFA in PBS.

The mice at P7, 2 and 6 weeks of age were intraperitoneally injected with 5-bromo-2′-deoxyuridine (BrdU) (95 mg/kg; Sigma Aldrich, St. Louis, MO, USA) 1 h before sacrifice to visualize the GCPs entering in S-phase, according to existing protocols (48, 49).

All the samples (with the exception of MBs employed for analyses of mRNAs and proteins) were cryoprotected in 30% sucrose in PBS and frozen at −80°C until use.

### Immunohistochemistry

Samples were embedded in Tissue-Tek OCT (Sakura Finetek, Torrance, CA, USA) and cut in free-floating 40-μm-thick sagittal sections at −25°C on a rotary cryostat.

To detect the BrdU incorporated into cellular DNA during cell proliferation, the sections were treated with 2N HCl for 45 min at 37°C to denature the nucleic acid, then with 0.1 M sodium borate buffer, pH 8.5, for 10 min. All the slices were permeabilized with 0.3% Triton X-100 in PBS and incubated overnight at 4°C with primary antibodies diluted in 3% normal donkey serum in PBS. The following primary antibodies were used: rat monoclonal antibody against BrdU (AbD Serotech, Raleigh, NC, USA; MCA2060; 1:400); rabbit monoclonal antibody against Ki67 (LabVision Corporation, Fremont, CA; clone SP6; 1:150); rabbit polyclonal antibody against cleaved (activated) caspase-3 (Cell Signaling Technology, Danvers, MA, USA; 9661; 1:100); mouse monoclonal antibodies raised against NeuN (Millipore, Temecula, CA, USA; MAB377; 1:300) or CD15 (Santa Cruz Biotechnology, Santa Cruz, CA, USA; sc-19648; 1:100); goat polyclonal antibody against NeuroD1 (R&D Systems, Minneapolis, MN, USA; AF2746; 1:200). To visualize the antigens, the samples were reacted with the appropriate secondary antibodies, all from Jackson ImmunoResearch (West Grove, PA, USA; 1:200): a donkey anti-rat TRITC-conjugated (BrdU), a donkey anti-rabbit Cy3-conjugated (Ki67, caspase-3), a donkey anti-mouse TRITC-conjugated (CD15) or conjugated to Alexa-488 (NeuN), a donkey anti-goat Cy3-conjugated (NeuroD1).

The samples were counterstained by Hoechst 33258 (Sigma-Aldrich; 1 mg/ml in PBS) to visualize the nuclei.

Digital images of the immunostained sections were collected with a TCS SP5 confocal laser scanning microscope (Leica Microsystems, Wetzlar, Germany). The CD15 immunolabeled MB slices were examined with an Olympus FV1200 laser scanning confocal microscope using a PlanApoN 60× Oil SC (NA = 1.40) Olympus objective. All the digital pictures were analyzed by the IAS software (Delta Sistemi, Rome, Italy).

### Quantification of Lesions and Cell Numbers

To evaluate the presence of preneoplastic lesions in mice of 2 or 6 weeks of age, we have examined for each cerebellum 12 sections, at 400 μm of distance, in order to allow a representative sampling. The lesions were identified by visualizing the GFP-positive GCPs.

The images of the lesions in cerebellar sagittal sections (**Figures 2A,B**) were obtained by an Olympus Optical (Tokyo, Japan) BX53 fluorescence microscope connected to a Spot RT3 camera (Diagnostic Instruments Inc., Sterling Heights, MI, USA).

BrdU^+^ or cleaved caspase-3^+^ cells in lesions were counted in the whole lesion area on at least five non-adjacent midsagittal sections per lesion. Planimetric measurements of lesion area were performed for the total extension of the lesion in each photomicrograph field by tracing the outline of the whole lesion on the digital picture and were measured with the IAS software. We analyzed the lesions present in all lesioned mice whose number for each genotype and time point is indicated in **Figure 2C**.

The proliferating (BrdU^+^), differentiating (NeuroD1^+^ or NeuN^+^), and apoptotic (cleaved caspase-3^+^) cells in the EGL of mice at P7 were measured on at least five non-adjacent midsagittal sections per mouse at the midpoint of the fifth, seventh, and ninth folia of each section, according to published protocols (12, 42); three mice for each genotype were analyzed. The EGL cell numbers were expressed as percentage ratio of proliferating, differentiating, or apoptotic cells to the total number of cells (labeled by Hoechst 33258), counted for the entire length of the EGL in each photomicrograph field.

Also in MBs, the proliferating (Ki67^+^), apoptotic (cleaved caspase-3^+^), or CD15-positive cell numbers were expressed as a percentage ratio to the total number of cells, labeled by Hoechst 33258. The cell counts were performed on about 40 randomly representative images, collected under the same parameters from five different tumors for each genotype.

### RNA Extraction, qRT-PCR

Total cellular RNA was extracted from *Ptch1*^+/−^*/Btg1*^+/+^ (*n* = 4) and *Ptch1*^+/−^*/Btg1*^−/−^ (*n* = 4) tumors with Trizol Reagent (Invitrogen, San Diego, CA, USA) and reverse-transcribed, as previously described (42). Real-time qPCR was carried out with a 7900HT System (Applied Biosystems, Foster City, CA, USA) using SYBR Green I dye chemistry in duplicate samples. The mRNA relative expression values, obtained by the comparative cycle threshold method (50), were normalized to expression levels of the *TATA-binding protein* gene, used as endogenous control. Specific qRT-PCR primers were designed by the Beacon Designer 7.1 software (Premier Biosoft International, Palo Alto, CA, USA) or by the BLAST software (Primer Blast NCBI: http://www.ncbi.nlm.nih.gov/tools/primer-blast) (51); their sequence is available on request.

### Western Blot Analysis

The *Ptch1*^+/−^*/Btg1*^+/+^ (*n* = 3) and *Ptch1*^+/−^*/Btg1*^−/−^ (*n* = 3) tumors were lysed into lysis buffer [50 mM Tris–HCl (pH 7.5), sodium deoxycholate 0.1%, 150 mM NaCl, 5 mM EDTA (pH 8.0), 10% glycerol, 1% NP40], containing 1 mM phenylmethylsulfonyl fluoride (PMSF), 10 μg/ml of leupeptin and aprotinin, 10 mM β-glycerophosphate, 4 mM NaF, 0.1 mM Na_3_VO_4_, 5 mM Na(C_3_H_7_COO), and sonicated. Proteins (80 μg per MB) were electrophoretically analyzed by sodium dodecyl sulfate (SDS)−10% polyacrylamide gel electrophoresis (PAGE) and transferred to nitrocellulose membranes; these were incubated for 2 h in blocking buffer {TBS [10 mM Tris–HCl (pH 8), 150 mM NaCl]−0.05% Tween−5% powdered milk} and then incubated overnight in the same buffer with the indicated primary antibodies.

The antibodies used for immunoblots were as follows: the rabbit polyclonal antibody anti-Prmt1 (07–404; Millipore; 1:500); the mouse monoclonal antibody anti-cyclin D1 (sc-20044; Santa Cruz Biotechnology; 1:200); the mouse monoclonal antibody anti-phospho-BAD (Ser99, sc-271963; Santa Cruz Biotechnology; 1:50); and the mouse monoclonal antibody DM1A anti-αTubulin (T6199; Sigma Aldrich; 1:10,000).

The densitometric analysis was performed using the Scan Analysis software (Biosoft, Cambridge, UK). Mean ± SEM densitometric values were obtained by averaging the percentage change of each MB relative to the control sample (one *Ptch1*^+/−^*/Btg1*^+/+^ tumor) and normalizing each sample value to the corresponding α-tubulin densitometric value, used as an endogenous control.

### Statistical Analysis

All statistical analyses were performed using StatView 5.0 software (SAS Institute, Cary, NC, USA).

The χ^2^-test was used to compare the MB incidence (Table 1) and the percentage of positivity for preneoplastic lesions (**Figure 2**) in mice of the different genotypes studied.

**Table 1:** Statistical analysis of medulloblastoma incidence and latency.

**Genotype**	***Ptch1^+/−^/Btg1^*WT*^***	***Ptch1^+/−^/Btg1*^+/−^**	***Ptch1^+/−^/Btg1^*KO*^***
Medulloblastoma (%)^*^	18 (34.62%)	21 (38.89%)	14 (42.42%)
Average latency (weeks)^†^	25.23 ± 2.71	24.57 ± 2.28	22.08 ± 2.13
No. of mice analyzed	52	54	33

* *No significant differences between the three genotypes studied (χ^2^-test)*.

† *No significant differences between the three genotypes studied (Student's t-test)*. *The three genotypes refer to the progeny of breeding between Patched1 heterozygotes (Ptch1^+/−^) and Btg1 knockout mice; all mouse genotypes are in the same genetic background, having been cross-mated for at least six generations*.

Data of MB time latency (Table 1) and number of lesions per cerebellum (**Figure 2**) were analyzed as average ± SEM and analyzed by the Student's *t*-test. Also the statistical analyses of mRNA and protein expression values were performed by Student's *t*-test.

**Figure 1:**
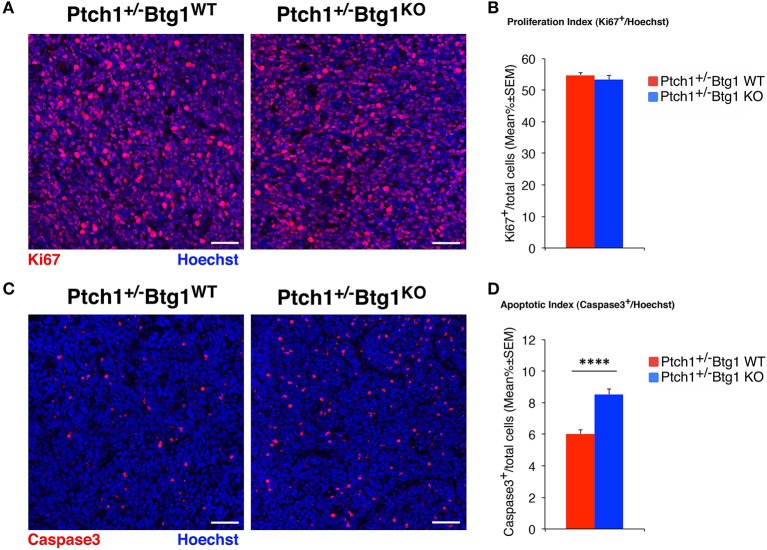
*B-cell translocation gene 1* (*Btg1*) ablation in *Patched1* heterozygous (*Ptch1*^+/−^) mice does not affect the proliferation of neoplastic cerebellar granule cell precursors (GCPs) but significantly increases their apoptosis. **(A)** Representative images by confocal microscopy showing the cycling cells (Ki67^+^, in red) in medulloblastomas (MBs) from *Ptch1*^+/−^*/Btg1*^*WT*^ and *Ptch1*^+/−^*/Btg1*^*KO*^ mice. Sections are counterstained with Hoechst 33258 to visualize the nuclei. Scale bar, 50 μm. **(B)** Analysis in *Ptch1*^+/−^*/Btg1*^*WT*^ and *Ptch1*^+/−^*/Btg1*^*KO*^ tumors of the number of proliferating GCPs, measured as mean ± SEM percentage ratio between number of Ki67^+^ cells and total number of cells (visualized by Hoechst 33258; proliferation index). Simple effects analysis: *p* = 0.8003, Mann–Whitney *U*-test. MBs analyzed: *n* = 5 for each genotype; total fields analyzed: *n* = 396. **(C)** Representative confocal images of *Ptch1*^+/−^*/Btg1*^*WT*^ and *Ptch1*^+/−^*/Btg1*^*KO*^ MBs stained for the apoptotic marker activated caspase-3 (red) and for the nuclei dye Hoechst (blue). Scale bars: 50 μm. **(D)** Quantification of mean ± SEM percentage ratio of cells positive for cleaved caspase-3 to the total number of cells (Hoechst-positive; apoptotic index) in MBs from *Ptch1*^+/−^*/Btg1*^*WT*^ and *Ptch1*^+/−^*/Btg1*^*KO*^ mice. Simple effects analysis: ^****^p < 0.0001, Mann–Whitney *U*-test. MBs analyzed: *n* = 5 for each genotype; total fields analyzed: *n* = 392.

The immunohistochemistry data of cell proliferation, differentiation, and apoptosis, as well as of CD15-positive cells (shown in Figures 1, **4**–**7**, respectively) were presented as mean% of total cells ± SEM. The experiments where the data were calculated as percentage of total cells were tested for the equality of variance between groups by the Levene's test; if equality was not satisfied, the data were analyzed with non-parametric tests, namely, Kruskal–Wallis test to analyze the main effects and Mann–Whitney *U*-test to analyze the simple effects (as for measurements of MB proliferation and apoptosis in Figure 1, of NeuN-positive cells in **Figure 5**, caspase-3-positive cells in **Figure 6**, and of CD15-positive cells in **Figure 7**). Data with equality of variance (i.e., proliferating cells in **Figure 4** and NeuroD1-positive cells in **Figure 5**) were analyzed using two-way ANOVA to test the main effects of the two genotypes (*Btg1* and *Ptch1*) on cell numbers. Individual between-group comparisons to test simple effects were carried out by Fisher's protected least significant difference (PLSD) ANOVA *post-hoc* test.

Moreover, the quantification by immunohistochemical analysis of apoptotic cells in preneoplastic lesions was obtained as mean ± SEM/mm^2^ of lesion and was statistically analyzed by Student's *t*-test (**Figure 3**).

All statistical tests were considered significant with *p* < 0.05.

## Results

### *B-Cell Translocation Gene 1* Ablation Does Not Enhance the Medulloblastoma Formation

It is known that the *Ptch1*^+/−^ mice develop spontaneous MBs arising from GCPs within 10 months of birth, with an incidence that is background-dependent (52). Moreover, we have observed that the ablation of *Btg1* increases the proliferation of GCPs, with consequent formation of a hyperplastic EGL (12).

Thus, we tested whether the *Btg1* deletion could affect the tumorigenic potential of GCPs. For this, we crossed the *Ptch1*^+/−^ mice with the *Btg1* knockout mice and analyzed the frequency of MB development in double-mutant mice during 1 year of life. Unexpectedly, we observed that in *Ptch1*^+/−^ background, the ablation of one or both alleles of *Btg1* did not significantly favor tumorigenesis, although in the double-mutant mice there was a trend to increase the MB incidence relative to the *Ptch1*^+/−^*/Btg1*^+/+^ mice (referred throughout this report to as *Ptch1*^+/−^*/Btg1*^*WT*^). In fact, the *Ptch1*^+/−^*/Btg1*^+/−^ and *Ptch1*^+/−^*/Btg1*^−/−^ (the latter hereafter referred to as *Ptch1*^+/−^*/Btg1*^*KO*^) mice developed MB with a frequency of 38.89 and 42.42%, respectively, relative to the MB incidence of *Ptch1*^+/−^*/Btg1*^*WT*^ mice (the latter incidence was 34.62%; *Ptch1*^+/−^*/Btg1*^+/−^ vs. *Ptch1*^+/−^*/Btg1*^*WT*^ *p* = 0.648; *Ptch1*^+/−^*/Btg1*^*KO*^ vs. *Ptch1*^+/−^*/Btg1*^*WT*^ *p* = 0.469; χ^2^-test; Table 1). Also the tumor latency in double-mutant mice, either *Ptch1*^+/−^*/Btg1*^+/−^ or *Ptch1*^+/−^*/Btg1*^*KO*^, did not significantly differ from that of control *Ptch1*^+/−^*/Btg1*^*WT*^ mice (*Ptch1*^+/−^*/Btg1*^+/−^ vs. *Ptch1*^+/−^*/Btg1*^*WT*^ *p* = 0.852; *Ptch1*^+/−^*/Btg1*^*KO*^ vs. *Ptch1*^+/−^*/Btg1*^*WT*^ *p* = 0.386; Student's *t*-test; Table 1).

To assess the effect of *Btg1* ablation on proliferation and apoptosis of neoplastic GCPs within the tumors, we carried out an immunohistochemical analysis of MB sections with the antibodies for Ki67, a protein expressed by cycling cells (53) or for the active proteolytic fragment of caspase-3, indicator of the apoptotic process (Figures 1A,C). Surprisingly, we found that the percentage of mitotic cells to the total number of cells (detected by Hoechst 33258; proliferation index) in MBs of *Ptch1*^+/−^*/Btg1*^*KO*^ mice was equivalent to that in tumors of *Ptch1*^+/−^*/Btg1*^*WT*^ mice (*p* = 0.8003; Mann–Whitney *U*-test; Figures 1A,B). Conversely, we observed in the *Ptch1*^+/−^*/Btg1*^*KO*^ MBs a highly significant increase in the percentage of caspase-3-positive cells to the total number of cells (apoptotic index), with respect to the *Ptch1*^+/−^*/Btg1*^*WT*^ tumors (*p* < 0.0001 and 42% increase; Mann–Whitney *U*-test; Figures 1C,D).

Next, we examined the effect of *Btg1* absence on the earliest stages of tumorigenesis by analyzing the premalignant lesions in the cerebella of *Ptch1*^+/−^*/Btg1*^+/+^*/Math1-GFP*^+^ and *Ptch1*^+/−^*/Btg1*^−/−^*/Math1-GFP*^+^ mice (referred throughout this report to as *Ptch1*^+/−^*/Btg1*^*WT*^*/Math1-GFP*^+^ and *Ptch1*^+/−^*/Btg1*^*KO*^*/Math1-GFP*^+^, respectively) sacrificed at different times, i.e., at 2 or 6 weeks. Two weeks of age is a stage corresponding to the end of proliferative development of GCPs within the EGL, when the lesions become morphologically detectable and are quite frequent (about 75%) (54); at 6 weeks of age, the EGL has disappeared but the lesions remain frequent, although only a limited number will develop into MB (47). The mouse model *Ptch1*^+/−^ is a powerful tool to identify the early stages in the MB development because within 2–6 months of birth, more than 50% of mice present at the surface of the cerebellum regions of ectopic cells, which represent a preneoplastic stage of tumorigenesis. These regions are usually nodular formations, extended into the fissures between the cerebellar lobules, and contain highly proliferating preneoplastic GCPs (pGCPs) (47, 55, 56). The pGCPs inside the lesions express the transcription factor *Math-1*, and then they can be identified in *Math1-GFP*^+^ mice by GFP expression (47, 56).

**Figure 2:**
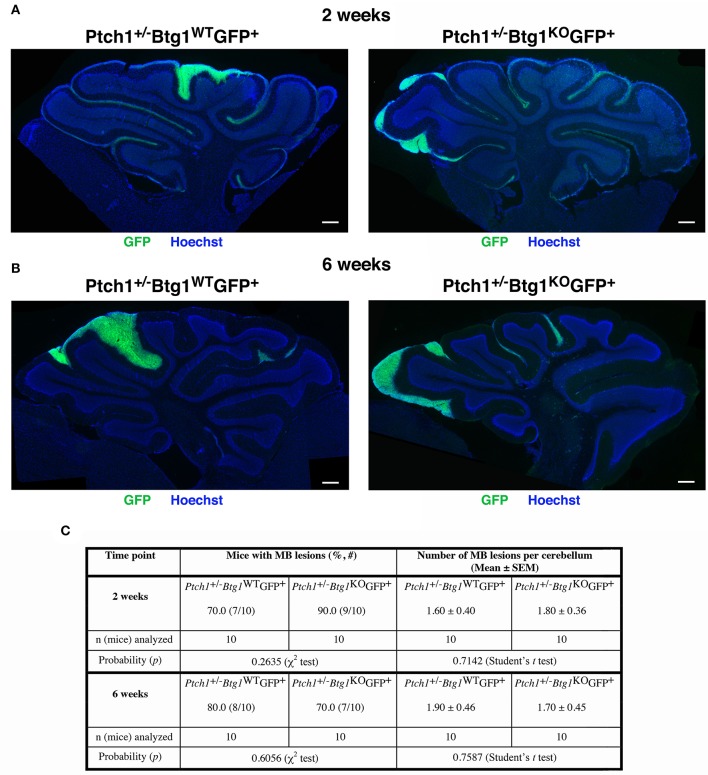
Deletion of the *B-cell translocation gene 1* (*Btg1*) gene in *Patched1* heterozygous (*Ptch1*^+/−^) background does not result in an enhancement of the frequency of mice with preneoplastic lesions nor does it affect the number of lesions per cerebellum. **(A,B)** Representative images of lesions in cerebellar sagittal sections from *Ptch1*^+/−^*/Btg1*^*WT*^*/Math1-GFP*^+^ and *Ptch1*^+/−^*/Btg1*^*KO*^*/Math1-GFP*^+^ mice at 2 **(A)** and 6 **(B)** weeks of age. Sections were stained with Hoechst 33258, and lesions were identified by the presence of green fluorescent protein (GFP)-positive preneoplastic cerebellar granule cell precursors (pGCPs). Scale bar, 300 μm. **(C)** Percentage of mice with lesions, frequency of lesions per cerebellum (obtained as average number in lesioned and non-lesioned mice) and number of mice analyzed for each genotype and time point; statistical analyses are indicated.

The screening of the cerebella of the asymptomatic *Ptch1*^+/−^*/Btg1*^*WT*^*/Math1-GFP*^+^ and *Ptch1*^+/−^*/Btg1*^*KO*^*/Math1-GFP*^+^ mice did not reveal differences between the two genotypes under study in the incidence of MB lesions (i.e., number of mice with lesions) or in the number of lesions per cerebellum neither at 2 weeks nor at 6 weeks of age (Figure 2). In particular, the ablation of the *Btg1* gene did not influence the expected frequency of GFP^+^ lesions in *Ptch1*^+/−^ mice at 6 weeks (*Ptch1*^+/−^*/Btg1*^*KO*^*/Math1-GFP*^+^ 70% frequency, and *Ptch1*^+/−^*/Btg1*^*WT*^*/Math1-GFP*^+^ 80% frequency, *p* = 0.6056, χ^2^-test; Figures 2B,C), which resulted in agreement with previous data (56).

**Figure 3:**
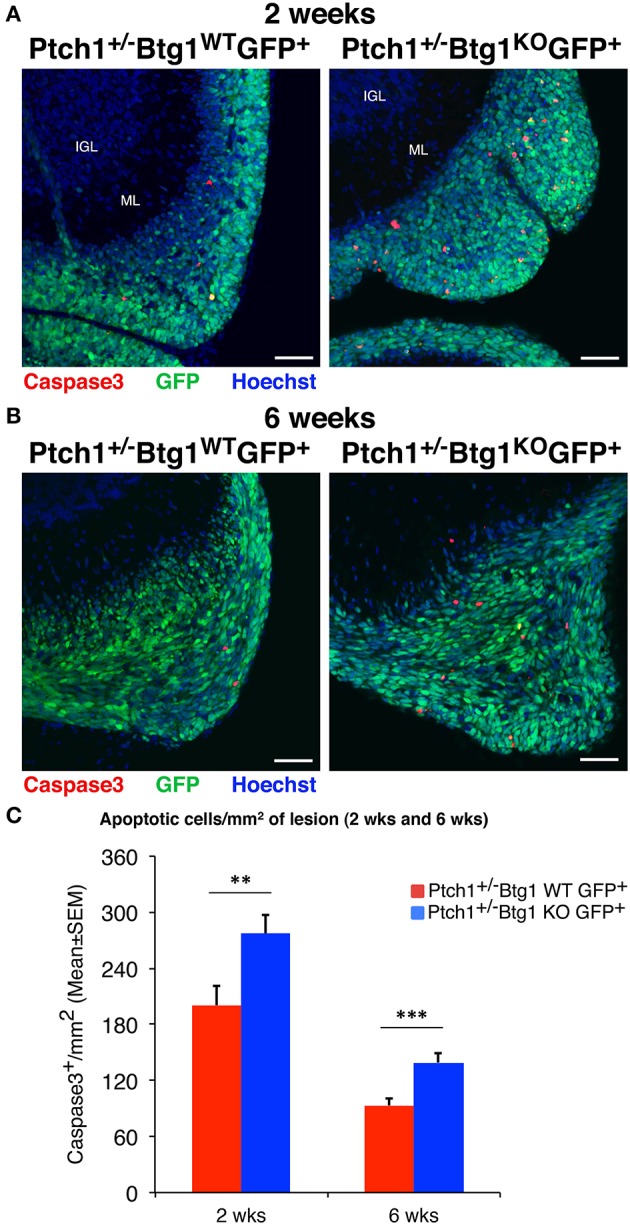
Ablation of *B-cell translocation gene 1* (*Btg1*) in *Patched1* heterozygous (*Ptch1*^+/−^) mice increases the apoptosis of preneoplastic cerebellar granule cell precursors (pGCPs). **(A,B)** Representative confocal images of apoptotic pGCPs, identified as cleaved caspase-3^+^ cells (in red), in preneoplastic lesions from *Ptch1*^+/−^*/Btg1*^*WT*^*/Math1-*green fluorescent protein (*GFP*)^+^ and *Ptch1*^+/−^*/Btg1*^*KO*^*/Math1-GFP*^+^ mice at 2 **(A)** and 6 **(B)** weeks of age. The pGCPs in the lesions are GFP^+^; the internal granule layer (IGL) and the molecular layer (ML) are visualized by Hoechst 33258 staining. Scale bar, 50 μm. **(C)** Apoptotic pGCPs were quantified as mean ± SEM/mm^2^ of cleaved caspase-3^+^ cells in the whole lesion area, defined by the presence of GFP^+^ cells. The lesions present in all lesioned mice were analyzed, whose number for each genotype and time point is indicated in Figure 2C. ^**^p < 0.01 or ^***^p < 0.001, Student's *t-*test. Total fields analyzed: *n* = 196 for 2 weeks, *n* = 151 for 6 weeks.

Then, we analyzed by immunohistochemistry the proliferation (after a 1-h pulse of BrdU) and the survival of pGCPs within the lesions. Similar to the MBs, no differences were detected between *Ptch1*^+/−^*/Btg1*^*WT*^*/Math1-GFP*^+^ and *Ptch1*^+/−^*/Btg1*^*KO*^*/Math1-GFP*^+^ mice at 2 and 6 weeks of age in the proliferative rate of pGCPs, expressed as the number of BrdU^+^ cells present in the GFP^+^ lesion area (data not shown). On the other hand, we observed a significant increase of apoptosis, measured as cleaved caspase-3^+^ cell number per lesion area, in *Ptch1*^+/−^*/Btg1*^*KO*^*/Math1-GFP*^+^ mice, relative to *Ptch1*^+/−^*/Btg1*^*WT*^*/Math1-GFP*^+^ mice, either at 2 weeks (*p* = 0.008 and 38% increase; Student's *t*-test; Figures 3A,C) or at 6 weeks of age (*p* = 0.0003 and 49% increase; Student's *t*-test; Figures 3B,C).

Together, these results indicate that the *Btg1* deletion in *Ptch1*^+/−^ mice did not enhance the neoplastic transformation of pGCPs and, then, the MB incidence, and suggest that this might be a consequence of a significant increase of apoptosis.

### *B-Cell Translocation Gene 1* Ablation Increases the Apoptosis of Granule Cell Precursors

Subsequently, we sought to assess the effects of the genetic ablation of *Btg1* in background *Ptch1*^+/−^ during the cerebellar development. To this aim, we measured the proliferation, differentiation, and survival of the GCPs within the EGL at P7, a stage corresponding to the age of highest expansion of GCPs.

**Figure 4:**
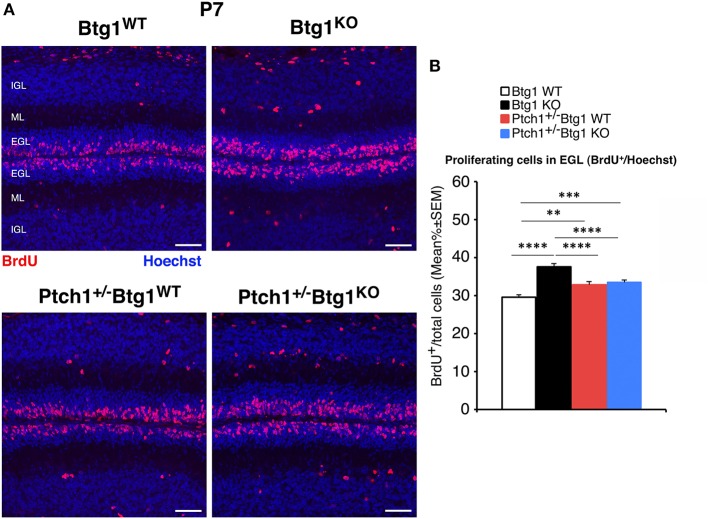
Genetic ablation of the *B-cell translocation gene 1* (*Btg1*) gene in *Patched1* heterozygous (*Ptch1*^+/−^) or in wild-type mice influences the proliferation of cerebellar granule cell precursors (GCPs) in the external granule layer (EGL) at postnatal day 7 (P7). **(A)** Representative confocal images of GCPs that have entered the cell cycle S-phase within the EGL of P7 mice of the four genotypes under study, identified as 5-bromo-2'-deoxyuridine (BrdU)-positive cells after a short pulse of BrdU (1 h). Sections are counterstained with Hoechst 33258 to visualize the EGL, the molecular layer (ML), and the internal granule layer (IGL). Scale bar, 50 μm. **(B)** Quantification of GCPs entering in the S-phase of cell cycle in the EGL of P7 mice with the indicated genotype, measured as mean ± SEM percentage ratio between the number of BrdU^+^ cells and the total number of cells (labeled by Hoechst 33258). The proliferative effect of *Btg1* deletion is more marked in wild-type than in *Ptch1*^+/−^ background [two-way ANOVA, *Btg1* ablation effect, *F*_(1,428)_ = 36.900, *p* < 0.0001; *Ptch1* ablation effect, *F*_(1,428)_ = 0.395, *p* = 0.5300, followed by analysis of simple effects: ^**^p < 0.01, ^***^p < 0.001, or ^****^p < 0.0001, Fisher's protected least significant difference (PLSD) ANOVA *post-hoc* test]. Three mice for each genotype were analyzed.

At P7, the percentage of proliferating GCPs to the total number of cells, identified by incorporation of BrdU after 1-h pulse, was significantly higher in *Ptch1*^+/+^*/Btg1*^*KO*^ mice than in *Ptch1*^+/+^*/Btg1*^*WT*^ mice (hereafter referred to as *Btg1*^*KO*^ and *Btg1*^*WT*^, respectively; 27.2% increase, *p* < 0.0001, Fisher's PLSD ANOVA *post-hoc* test; Figures 4A,B), as expected (12). Also in *Ptch1*^+/−^*/Btg1*^*WT*^ mice, the GCP proliferation was increased by constitutive activation of the Shh pathway (*Ptch1*^+/−^*/Btg1*^*WT*^ vs. *Btg1*^*WT*^ *p* = 0.0012, 11.1% increase, Fisher's PLSD ANOVA *post-hoc* test; Figures 4A,B). The latter increase of proliferation agrees with previous observations (42) and is significantly lower than that induced by *Btg1* deletion alone (*Ptch1*^+/−^*/Btg1*^*WT*^ vs. *Btg1*^*KO*^ *p* < 0. 0001, Fisher's PLSD ANOVA *post-hoc* test; Figures 4A,B). Notably, no positive synergy was observed between the Btg1 and Shh pathways since the ablation of *Btg1* did not increase the percentage of proliferating GCPs above the level induced by the constitutively activated Shh signaling pathway (*Ptch1*^+/−^*/Btg1*^*KO*^ vs. *Ptch1*^+/−^*/Btg1*^*WT*^ *p* = 0.5737, Fisher's PLSD ANOVA *post-hoc* test; Figures 4A,B).

**Figure 5:**
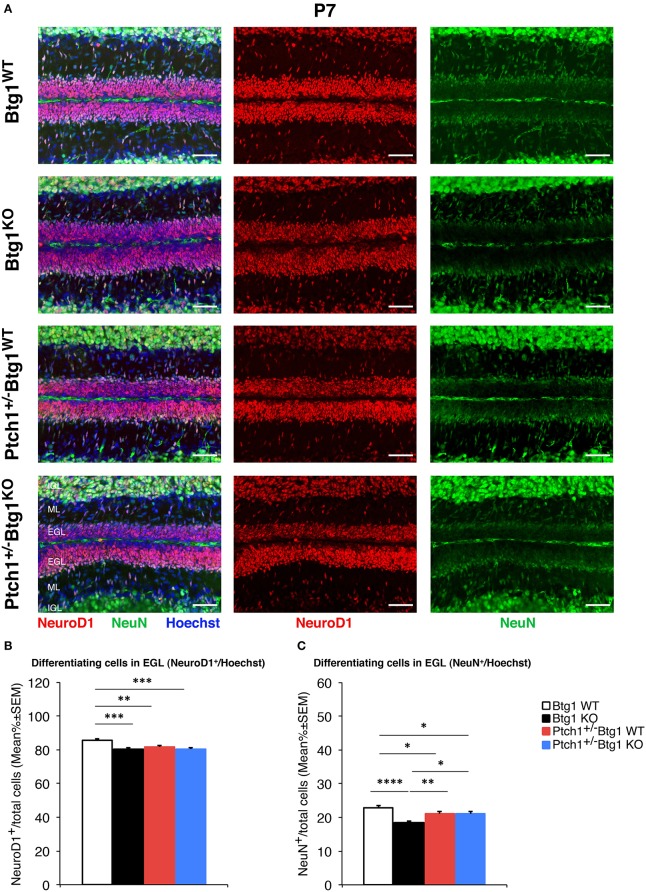
*B-cell translocation gene 1* (*Btg1*) ablation in *Patched1* heterozygous (*Ptch1*^+/−^) or wild-type mice reduces the differentiation of cerebellar granule cell precursors (GCPs) in the external granule layer (EGL) at postnatal day 7 (P7). **(A)** Representative images by confocal microscopy showing the GCPs in the EGL of P7 mice of the four genotypes indicated, labeled with the early and the late differentiation markers NeuroD1 (red) and NeuN (green), respectively. Sections are counterstained with Hoechst 33258 to visualize the EGL and the molecular layer (ML) and internal granule layer (IGL). Scale bar, 50 μm. **(B,C)** Quantitative analysis of GCPs expressing NeuroD1 **(B)** or NeuN **(C)** in the EGL of P7 mice of the four genotypes under study. Data are mean ± SEM percentage ratio between the number of NeuroD1^+^ or NeuN^+^ GCPs and the total number of GCPs within the EGL, labeled by Hoechst 33258. **(B)** Two-way ANOVA: *Btg1* ablation effect, *F*_(1,352)_ = 11.434, *p* = 0.0008; *Ptch1* ablation effect, *F*_(1,352)_ = 3.908, *p* = 0.0488. Simple effects analysis: ^**^p < 0.01 or ^***^p < 0.001, Fisher's protected least significant difference (PLSD) ANOVA *post-hoc* test. Three mice for each genotype were analyzed. **(C)** Main effect analysis by Kruskal-Wallis [d.f. 3] *H* = 26.444, *p* < 0.0001, followed by analysis of simple effects: ^*^p < 0.05, ^**^p < 0.01, or ^****^p < 0.0001, Mann–Whitney *U*-test. Mice analyzed: *n* = 3 for each genotype; total fields analyzed: *n* = 356.

Next, we measured the differentiation of GCPs by labeling these cells with the neural markers NeuroD1 and NeuN (Figure 5). NeuroD1 is highly expressed in early post-mitotic GCPs and is implicated in their differentiation (57), while NeuN labels fully differentiated granule neurons (58). At P7, we observed a significant decrease in the percentage of NeuroD1^+^ GCPs in all conditions of augmented proliferation (*Btg1*^*WT*^ vs. *Btg1*^*KO*^ *p* = 0.0001; *Btg1*^*WT*^ vs. *Ptch1*^+/−^*/Btg1*^*WT*^ *p* = 0.0063; *Btg1*^*WT*^ vs. *Ptch1*^+/−^*/Btg1*^*KO*^ *p* = 0.0002; Fisher's PLSD ANOVA *post-hoc* test; Figures 5A,B). Furthermore, no difference was observed in NeuroD1 expression between mice with the genetic ablation of *Btg1* alone or combined with the deletion of the *Ptch1* allele, indicating that no evident synergy occurred between the *Btg1*-null and the *Ptch1*^+/−^ background (Figures 5A,B). Similarly, the percentage of NeuN-positive GCPs was significantly lower in all groups relative to the *Btg1*^*WT*^ mice, with a slightly more pronounced decrease in the *Btg1*^*KO*^ mice (*Btg1*^*WT*^ vs. *Btg1*^*KO*^ *p* < 0.0001; *Btg1*^*WT*^ vs. *Ptch1*^+/−^*/Btg1*^*WT*^ *p* = 0.0200; *Btg1*^*WT*^ vs. *Ptch1*^+/−^*/Btg1*^*KO*^ *p* = 0.0117; Mann–Whitney *U*-test; Figures 5A,C). *Ptch1*^+/−^*/Btg1*^*WT*^ and *Ptch1*^+/−^*/Btg1*^*KO*^ mice presented equivalent percentages of NeuN^+^ GCPs, significantly higher than that in *Btg1*^*KO*^ mice (*Ptch1*^+/−^*/Btg1*^*WT*^ vs. *Btg1*^*KO*^ *p* = 0.0048; *Ptch1*^+/−^*/Btg1*^*KO*^ vs. *Btg1*^*KO*^ *p* = 0.0154; Mann–Whitney *U*-test; Figures 5A,C). All this indicates that the ablation of *Btg1*, although associated to a decrease of differentiation, did not interfere with the inhibition of differentiation exerted by the *Ptch1*^+/−^ genotype.

**Figure 6:**
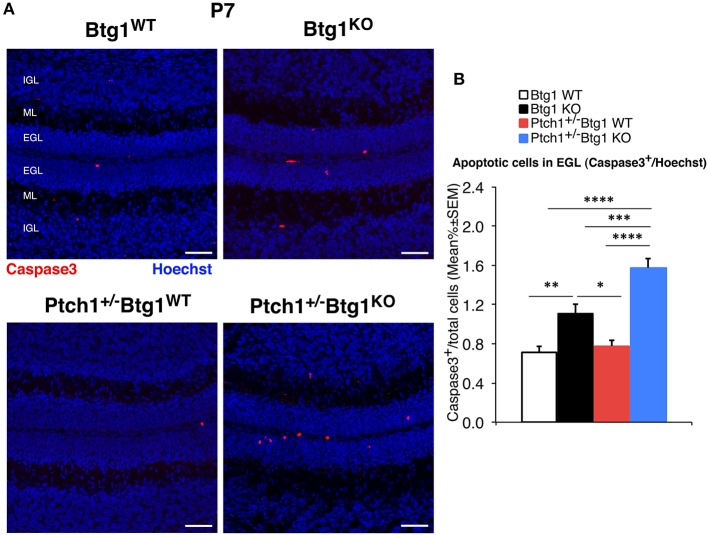
Deletion of the *B-cell translocation gene 1* (*Btg1*) gene promotes the apoptosis of cerebellar granule cell precursors (GCPs) within the external granule layer (EGL) in postnatal day 7 (P7) *Patched1* heterozygous (*Ptch1*^+/−^) mice more than in wild-type mice. **(A)** Representative confocal images of apoptotic GCPs, identified as cleaved caspase-3^+^ cells, in the EGL of P7 mice of the four genotypes indicated. Sections were stained with Hoechst 33258 to visualize the EGL, the molecular layer (ML) and the internal granule layer (IGL). Scale bar, 50 μm. **(B)** Apoptotic GCPs in the EGL of P7 mice of the four genotypes under study were quantified as mean ± SEM percentage ratio between the number of cleaved caspase-3^+^ GCPs (in red) and the total number of GCPs within the EGL (Hoechst-positive). *Btg1* knockout significantly increases the percentage of GCPs undergoing apoptosis either in *Ptch1*^+/−^ or in wild-type mice, with a greater fraction of apoptotic GCPs in double-mutant mice (main effect analysis by Kruskal–Wallis [d.f. 3] *H* = 62.761, *p* < 0.0001, followed by analysis of simple effects: ^*^p < 0.05, ^**^p < 0.01, ^***^p < 0.001, or ^****^p < 0.0001, Mann–Whitney *U*-test). Mice analyzed: *n* = 3 for each genotype; total fields analyzed: *n* = 462.

Then, we analyzed the survival of GCPs by detection of cleaved caspase-3^+^ cells in the EGL. At P7, *Btg1* knockout significantly increased the percentage of GCPs undergoing apoptosis either in *Ptch1* wild-type mice (*Btg1*^*KO*^ vs. *Btg1*^*WT*^ *p* = 0.0011 and 55% increase; Mann–Whitney *U*-test; Figures 6A,B), as expected (12), or in *Ptch1*^+/−^ mice (*Ptch1*^+/−^*/Btg1*^*KO*^ vs. *Btg1*^*WT*^ *p* < 0.0001 and 122% increase; Mann–Whitney *U*-test; Figures 6A,B). Interestingly, although the ablation of one *Ptch1* allele alone did not affect the apoptosis of the GCPs (*Ptch1*^+/−^*/Btg1*^*WT*^ vs. *Btg1*^*WT*^ *p* = 0.3723; Mann–Whitney *U*-test; Figures 6A,B), the combined genetic deletion of *Btg1* gene significantly augmented the percentage of caspase-3^+^ GCPs (*Ptch1*^+/−^*/Btg1*^*KO*^ vs. *Ptch1*^+/−^*/Btg1*^*WT*^ *p* < 0.0001; Mann–Whitney *U*-test; Figures 6A,B), far above the level induced by *Btg1* deletion alone (*Ptch1*^+/−^*/Btg1*^*KO*^ vs. *Btg1*^*KO*^ *p* = 0.0002; Mann–Whitney *U*-test; Figures 6A,B).

Overall, this indicates that during cerebellar development, the ablation of *Btg1* in *Ptch1* heterozygous mice does not influence the proliferation and differentiation processes, which are only dependent on the constitutive activation of the Shh pathway, but significantly increases the percentage of caspase-3-positive GCPs, suggesting the existence of a synergy between the Btg1 and Shh pathways in the apoptotic process.

### *B-Cell Translocation Gene 1* Ablation Induces Prmt1-Mediated Apoptosis and Increases Stemness of Tumor Cells

**Figure 7:**
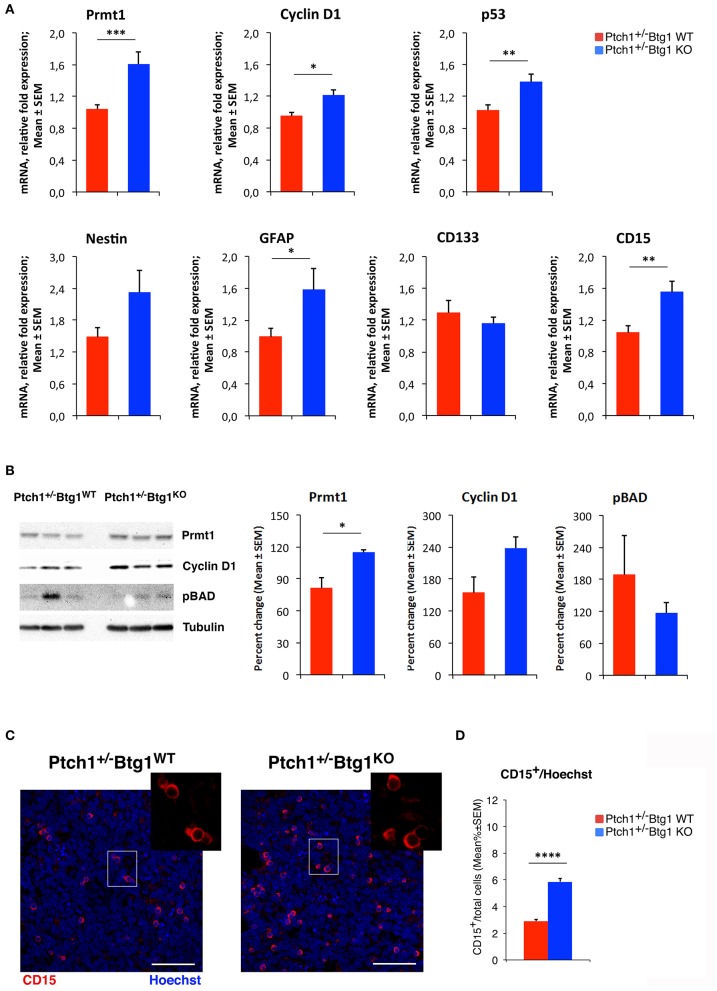
*B-cell translocation gene 1* (*Btg1*) ablation in *Patched1* heterozygous (*Ptch1*^+/−^) mice induces protein arginine methyltransferase 1 (Prmt1) expression and increases stemness in medulloblastoma (MB) cells. **(A)** Real-time PCR analysis of mRNA obtained from *Ptch1*^+/−^*/Btg1*^*WT*^ and *Ptch1*^+/−^*/Btg1*^*KO*^ MBs. Four tumors per genotype were analyzed. Average ± SEM values are from three to four independent analyses of each tumor and are shown as fold change relative to the control sample (one *Ptch1*^+/−^*/Btg1*^*WT*^ MB), which was set to unit. *TBP* mRNA was used to normalize data. ^*^p < 0.05, ^**^p < 0.01, or ^***^p < 0.001, Student's *t-*test. **(B)** Immunoblotting and densitometry analysis with anti-Prmt1, anti-cyclin D1, or anti-phospho-BAD antibodies in *Ptch1*^+/−^*/Btg1*^*WT*^ and *Ptch1*^+/−^*/Btg1*^*KO*^ MBs. Three tumors per genotype were analyzed. Mean ± SEM densitometric values were obtained by averaging the percentage change of each MB relative to the control sample (one *Ptch1*^+/−^*/Btg1*^*WT*^ MB) and normalizing each sample value to the corresponding α-tubulin densitometric value, used as endogenous control. ^*^p < 0.05, Student's *t-*test. **(C)** Representative images by confocal microscopy showing the tumor stem cells (CD15^+^, in red) in MBs from *Ptch1*^+/−^*/Btg1*^*WT*^ and *Ptch1*^+/−^*/Btg1*^*KO*^ mice. Nuclei were stained with Hoechst 33258. Scale bar, 50 μm. The white box area is shown with 2.2× magnification. **(D)** Quantification of mean ± SEM percentage ratio of cells positive for CD15 to the total number of cells, labeled by Hoechst 33258, in *Ptch1*^+/−^*/Btg1*^*WT*^, and *Ptch1*^+/−^*/Btg1*^*KO*^ MBs. Simple effects analysis: ^****^p < 0.0001, Mann–Whitney *U*-test. MBs analyzed: *n* = 5 for each genotype; total fields analyzed: *n* = 400.

To investigate the molecular mechanisms of increased apoptosis following the genetic ablation of *Btg1*, we first analyzed by qRT-PCR the expression levels of three components of the apoptotic pathway, namely, B-cell lymphoma 2 (Bcl-2), which is an antiapoptotic protein, p53 upregulated modulator of apoptosis (Puma), and Bcl-2-associated X protein (Bax), identified as apoptotic inducers. No significant changes of these mRNAs were detected in *Ptch1*^+/−^*/Btg1*^*KO*^ tumors with respect to *Ptch1*^+/−^*/Btg1*^*WT*^ tumors (data not shown). Very recently, the involvement of Prmt1 in the apoptosis of tumor cells has been demonstrated, although with opposite roles in different types of cancer (59, 60). Since Prmt1 is a known molecular partner of Btg1, we investigated the *Prmt1* level in the MBs and we observed a significant increase of its expression in *Ptch1*^+/−^*/Btg1*^*KO*^ tumors relative to *Ptch1*^+/−^*/Btg1*^*WT*^ tumors (54.4% increase, *p* = 0.0021; Student's *t*-test; Figure 7A). Moreover, we analyzed the mRNA level of *cyclin D1*, target gene of Btg1, and we found that in *Ptch1*^+/−^*/Btg1*^*KO*^ MBs the *cyclin D1* expression was significantly higher than in *Ptch1*^+/−^*/Btg1*^*WT*^ MBs (*p* = 0.0117; Student's *t*-test; Figure 7A). In our experience, this increase should be associated to great changes in cellular proliferation (12); however, *Ptch1*^+/−^*/Btg1*^*KO*^ MBs show a proliferation index equivalent to that of *Ptch1*^+/−^*/Btg1*^*WT*^ MBs (see Figures 1A,B). Yet, we also observed an increased *p53* expression within the tumors lacking the *Btg1* gene (*Ptch1*^+/−^*/Btg1*^*KO*^ vs. *Ptch1*^+/−^*/Btg1*^*WT*^ *p* = 0.0070; Student's *t*-test; Figure 7A), which, given the proapoptotic action of p53, may explain the lack of increase of proliferation in MBs.

We have previously demonstrated that in the neurogenic niches, Btg1 controls the quiescence and self-renewal of the stem cells (11, 29), then by qRT-PCR, we also evaluated in the MBs the mRNA levels of some markers of brain tumor stem cells, such as nestin, glial fibrillary acidic protein (GFAP), CD133, and CD15 (38, 61–68). In *Ptch1*^+/−^*/Btg1*^*KO*^ MBs, we observed an increase of the levels of *nestin* and *GFAP* mRNAs relative to the *Ptch1*^+/−^*/Btg1*^*WT*^ MBs, with the *GFAP* expression attaining statistical significance (*p* = 0.0467; Student's *t*-test; Figure 7A). Furthermore, we recorded a statistically significant increase of *CD15* expression in *Ptch1*^+/−^*/Btg1*^*KO*^ tumors with respect to *Ptch1*^+/−^*/Btg1*^*WT*^ tumors (*p* = 0.0057; Student's *t*-test; Figure 7A), while no changes in mRNA level of *CD133* were detected between the tumors of the two different genotypes under study (*p* = 0.2455; Student's *t*-test; Figure 7A).

Finally, no significant differences were observed for the main downstream Shh-responsive genes, namely, *Gli1, Gli2, Ptch1*, and *N-Myc*, in *Ptch1*^+/−^*/Btg1*^*KO*^ MBs relative to *Ptch1*^+/−^*/Btg1*^*WT*^ MBs (data not shown), indicating that the deletion of *Btg1* does not interfere with Shh signaling in cerebellar tumors.

Also, at the protein level, we found a significant increase of Prmt1 in *Ptch1*^+/−^*/Btg1*^*KO*^ MBs with respect to *Ptch1*^+/−^*/Btg1*^*WT*^ MBs (*p* = 0.028; Student's *t*-test; Figure 7B). Moreover, we observed an increase of the level of cyclin D1 protein in *Ptch1*^+/−^*/Btg1*^*KO*^ tumors relative to the *Ptch1*^+/−^*/Btg1*^*WT*^ tumors, without attaining statistical significance (*p* = 0.089; Student's *t*-test; Figure 7B).

It is known that the methylation of the proapoptotic BAD protein at Arg-94 and Arg-96 catalyzed by Prmt1 inhibits Akt-mediated BAD phosphorylation at Ser-99, thus counteracting the Akt-dependent survival signaling and inducing the apoptotic process with concomitant activation of caspase-3 (44). Then, we analyzed the protein level of BAD phosphorylated at Ser-99 (pBAD) in MBs of *Ptch1*^+/−^*/Btg1*^*KO*^ and *Ptch1*^+/−^*/Btg1*^*WT*^ mice and we observed a decrease, although not significant, in BAD phosphorylation in tumors lacking the *Btg1* gene (*p* = 0.397; Student's *t*-test; Figure 7B). This suggests that the *Btg1* ablation could increase the apoptotic process by inducing the Prmt1-dependent methylation of the BAD protein.

We also analyzed the expression of GFAP protein in the MBs of both genotypes by measuring the area of GFAP staining detected by immunohistochemistry (see Supplementary Material); consistently with the mRNA expression, we observed a significant increase of GFAP protein in *Ptch1*^+/−^*/Btg1*^*KO*^ vs. *Ptch1*^+/−^*/Btg1*^*WT*^ MBs (*p* = 0.0074; Mann–Whitney *U*-test; Figure S1).

The increase of expression of *CD15*, a marker of brain tumor stem cells, detected by qRT-PCR in the *Ptch1*^+/−^*/Btg1*^*KO*^ MBs compared with *Ptch1*^+/−^*/Btg1*^*WT*^ MBs (Figure 7A), was confirmed at protein level by immunohistochemical analysis of tumor sections from MBs of the two different genotypes under study (Figures 7C,D). In MBs lacking the *Btg1* gene, we observed a two-fold increase of the percentage of CD15-positive cells with respect to *Ptch1*^+/−^*/Btg1*^*WT*^ tumors (*p* < 0.0001; Mann–Whitney *U*-test; Figures 7C,D), with a greater tendency of CD15^+^ cells to aggregate into clusters.

Noteworthy, by immunohistochemical analysis, we also evaluated in tumors and preneoplastic lesions of *Ptch1*^+/−^*/Btg1*^*WT*^ and *Ptch1*^+/−^*/Btg1*^*KO*^ mice the expression of Sox2, a marker for tumor stem cells in Shh-induced MB (69), without finding any difference (data not shown).

## Discussion

In childhood, the primary cause of cancer-related death is brain tumors. Among these, MB, a highly malignant neoplasia developing in the cerebellum, is the most common, representing about 20% of all pediatric cerebral tumors (70, 71). The standard therapy for MB, which includes preliminary surgery followed by radiation and chemotherapy, allows a 5-years survival rate of 60% due to the frequent recurrences with a fatal outcome (72). Moreover, the toxicity of the chemotherapeutic drugs and the non-specificity of the radiation cause permanent neurocognitive damages to the young patients (73). So, it is necessary to develop innovative treatment strategies based on the use of more specific and less toxic molecules.

In this regard, we have already identified two molecules that act as tumor suppressors of MB, PC3^TIS21/BTG2^ (40, 41) and Cxcl3 (74). Then, in this report, we investigated the role in the MB pathogenesis of the *PC3*^*TIS*21/*BTG*2^-related gene *Btg1*, which we have previously shown to be required for the development and function of the cerebellum by operating basically as a negative regulator of the proliferation of cerebellar cell precursors (GCPs) through inhibition of cyclin D1 (12).

In this report, however, we observe that no significant change in tumor frequency of *Ptch1*^+/−^ mice occurs after deletion of *Btg1*. This is quite surprising given the increase of proliferation of cerebellar GCPs caused by the ablation of *Btg1* in *Patched1* wild-type background; moreover, *Btg1* has been shown to act as oncosuppressor in MB cells which strongly inhibits the proliferation (12) and also in several types of tumors (14, 29).

We expected that the increase of proliferation of GCPs observed in the EGL of *Ptch1*^+/+^*/Btg1*^*KO*^ mice at P7 caused by *Btg1* deletion would lead in *Ptch1*^+/−^*/Btg1*^*KO*^ cerebella to an increase of *Ptch1*^+/−^ cells, which are prone to tumor development. Consequently, we would have expected an increase of tumor frequency. However, surprisingly, in GCPs at P7, no proliferative synergy occurs in double-mutant *Ptch1*^+/−^*/Btg1*^*KO*^ vs. single mutant *Ptch1*^+/−^*/Btg1*^*WT*^ cells—although either the *Patched1* or the *Btg1* mutation alone is able to significantly increase proliferation—suggesting that the two proliferative pathways are independent. No increase of proliferation is observed also in *Ptch1*^+/−^*/Btg1*^*KO*^ vs. *Ptch1*^+/−^*/Btg1*^*WT*^ tumor cells. However, as we have previously shown that Btg1 inhibits selectively cyclin D1 (12), we observe here that the ablation of *Btg1* is associated to an increase of *cyclin D1* expression (though attaining statistical significance only for the transcript) in MB tumor cells, indicating that the proliferative system is activated, despite the lack of increased proliferation of *Ptch1*^+/−^*/Btg1*^*KO*^ GCPs or tumor cells.

A possibility that may explain the lack of increase of proliferation and tumorigenesis is that the concomitant great increase of apoptosis occurring in *Ptch1*^+/−^*/Btg1*^*KO*^ double-mutant precursor cells, relative to single mutant cells, may prevent their expansion and thus prevent an increase of tumor frequency in double-mutant cerebella.

Moreover, the *Btg1* deletion, but not the *Patched1* mutation alone, leads to a significant increase of apoptosis, relative to *Btg1*^*WT*^, while the two mutations together (*Ptch1*^+/−^*/Btg1*^*KO*^) synergize significantly, relative to *Btg1* deletion alone, given that the *Btg1*^*KO*^ apoptotic GCPs are significantly more in *Ptch1*^+/−^ background than in *Patched1* wild-type background. Consistently, we have previously shown that in *Ptch1*^+/−^ mice, no change in apoptosis occurs (42). Also in MB lesions and in tumor cells, i.e., in *Ptch1*^+/−^ background, the deletion of *Btg1* highly increases apoptosis.

Such synergy of the *Btg1* mutation with the *Patched1* mutation may depend on the fact that each mutation could drive the cell into a permissive state for apoptosis, which is fully activated only when both mutations are present. In fact, although the activation of the Shh pathway has been associated to greater survival, nevertheless, an active pathway induces the transition of the proapoptotic protein Bax to a potentially active state ready to be triggered by an apoptotic stimulus (75). This may not be in contrast with our data, considering that the inactivation of Btg1 leads to a condition of greater and/or deregulated proliferation that may perturb or revert the prosurvival action of the Shh pathway. In fact, it is known that the loss of a negative regulator of proliferation, as for instance pRb (76), causes misregulation of the proliferative pathways, leading to apoptosis. Moreover, we have previously observed that the knockout of *Btg1* increases the fraction of p53-positive as well as of caspase-3-positive cells in the dentate gyrus (11). Consistently, here we observe that *p53* expression increases also in MB tumor cells ablated of *Btg1*, relative to MB tumor cells with *Btg1* gene, which is compatible with an increase of apoptosis [see (77) for a review of the proapoptotic role of p53], suggesting that an increase of p53 is a cause for reduced survival. Thus, the loss of *Btg1* may synergize with the Shh/Patched1 pathway to enhance apoptosis and may have targets either in common or distinct from this pathway.

In molecular terms, we also find that the deletion of *Btg1* increases the expression of Prmt1. It is known that Prmt1 activates the proapoptotic BAD gene by methylating the BAD protein, in this way maintaining the BAD protein in a dephosphorylated active state, which enables it to bind Bcl-X_L_ and to prevent its antiapoptotic function (44). In fact, we observe a decrease of the levels of phosphorylated BAD protein in MBs with *Btg1*^*KO*^ background, though not attaining statistical significance, probably because of the variability between tumors. Further analyses will be necessary to examine this mechanism.

Notably, we observe an increase in *Btg1* knockout MBs of cells positive for CD15 that labels tumor stem cells in brain tumors as well as in MBs (64–67). These tumor cells are known to remain in a quiescent state that reduces the possibility of successful therapy with antiproliferative strategies (78). We observed an increase also of *GFAP*, which labels about 5% of precursor cells in *Ptch1*^+/−^ MBs (67) and is a transcriptional target of Prmt1 (79). This finding in MB is in line with what is observed in the adult neurogenic niches, i.e., in the dentate gyrus of the hippocampus and the subventricular zone, where the loss of cell cycle control consequent to the absence of *Btg1* acutely elicits an uncontrolled proliferation of stem/progenitor cells, followed by an age-dependent decrease of their proliferative capacity and by quiescence (11). Thus, we can speculate that such an increase of CD15^+^ cells in the *Ptch1*^+/−^*/Btg1*^*KO*^ model may be ascribed to an activation of existing tumor stem cells—possibly triggered by the high level of apoptosis of tumor cells—followed by their entry into quiescence. Future analyses will be necessary to evaluate whether this increase of tumor stem cells may cause a greater resistance to therapy of the *Btg1*-null MBs, especially in consideration of the data by Ward et al. (67), indicating a stronger tumor-initiating ability of CD15^+^ tumor cells orthotopically transplanted from Shh-type MBs.

In conclusion, our findings, although showing that the ablation of *Btg1* does not increase MB frequency in a *Ptch1*^+/−^ model, do not exclude that Btg1 acts as an MB suppressor, given the large increase of apoptosis following deletion of *Btg1* that may counteract and hide any increase of proliferation and tumorigenesis. In fact, *Btg1* expression appears to regulate the balance between proliferation and apoptosis in MB tumor formation. *Btg1* is also deregulated in many tumor types (see *Introduction*); in human MB there is, on average, no significant change of *Btg1* expression relative to control cerebellum samples, but, remarkably, some MB samples show ample deregulation of expression (up to 40%; *ONCOMINE* database: www.oncomine.org) (80). Additionally, mutations of *Btg1* in human tumors are frequent (COSMIC database: http://cancer.sanger.ac.uk) (81); this aspect should be further tested in human as well as in mouse MB. Moreover, in view of the strong increase observed in our *Btg1*-null MB mouse model of CD15^+^ tumor stem cells, which can be at the origin of tumor regrowth after years, future investigation may evaluate whether the *Btg1* gene is implicated in MB relapse.

## Data Availability Statement

The datasets generated for this study are available on request to the corresponding authors.

## Ethics Statement

This animal study was reviewed and approved by Italian Ministry of Health that approved these experiments on mice with authorization 193/2015-PR.

## Author Contributions

MC and FT designed the research, analyzed the data, and wrote the paper. MC, GD'A, and LM performed the research. All authors read and approved the final version of the manuscript.

### Conflict of Interest

The authors declare that the research was conducted in the absence of any commercial or financial relationships that could be construed as a potential conflict of interest.

## Abbreviations

ANOVA: analysis of variance
BAD: Bcl-2 antagonist of cell death
BrdU: 5-bromo-2′-deoxyuridine
Btg1: B-cell translocation gene 1
CD15: cluster of differentiation 15
EGL: external granule layer
GCPs: cerebellar granule cell precursors
GFAP: glial fibrillary acidic protein
GFP: green fluorescent protein
IGL: internal granule layer
KO: knockout
Math1: mouse atonal homolog 1
MB: medulloblastoma
ML: molecular layer
NeuN: neuronal nuclei
pGCPs: preneoplastic cerebellar granule cell precursors
PLSD: protected least significant difference
P7: postnatal day 7
Prmt1: protein arginine methyltransferase 1
Ptch1: Patched1 gene
SEM: standard error of the mean
Shh: Sonic hedgehog
TRITC: tetramethylrhodamine
WT: wild-type.
