# Supplementary material for: Deletion of Btg1 Induces Prmt1-Dependent Apoptosis and Increased Stemness in Shh-Type Medulloblastoma Cells Without Affecting Tumor Frequency
Source: Front Oncol. 2020 Mar 13;10:226. doi: 10.3389/fonc.2020.00226 (PMC7082329; doi:10.3389/fonc.2020.00226)
Supplement: Supplementary file 1 [file Data_Sheet_1.pdf]

Supplementary Material

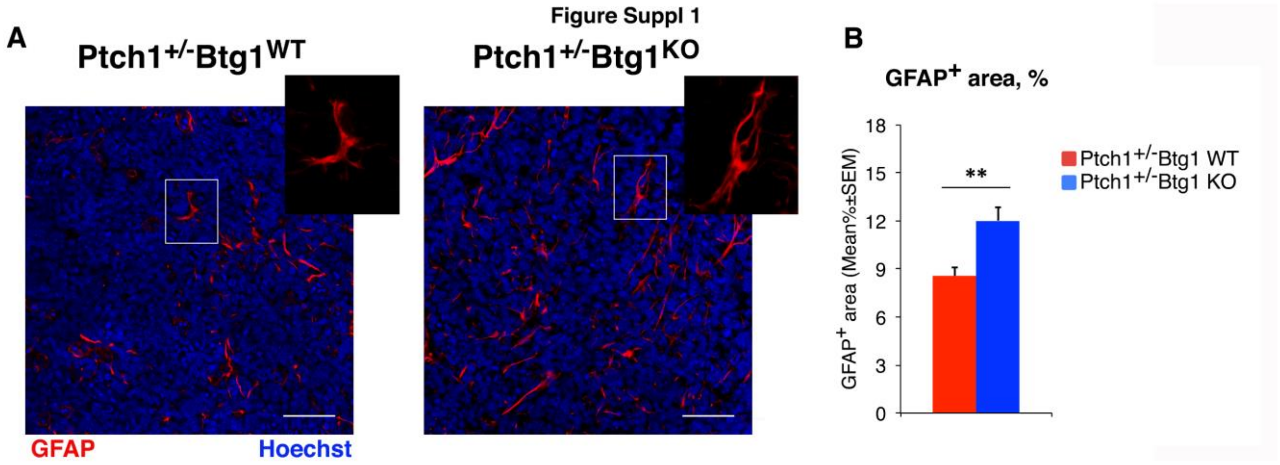

Figure S1. *Btg1* ablation in *Ptch1*$^{+/–}$ mice significantly increases glial fibrillary acidic protein (GFAP) expression in medulloblastomas (MBs). (A) Representative confocal images of the staining with the brain tumor stem cells marker GFAP (red) and nuclear marker Hoechst 33258 (blue) of MBs from *Ptch1*$^{+/–}$/*Btg1*^{WT} and *Ptch1*$^{+/–}$/*Btg1*^{KO} mice. Scale bar, 50 µm. The white box areas are shown with 2.2× digital magnification. **(B)** The GFAP density was semi-quantified as mean% ± SEM of area of GFAP staining in either *Ptch1*$^{+/–}$/*Btg1*^{WT} or *Ptch1*$^{+/–}$/*Btg1*^{KO} medulloblastomas. ** $p < 0.01$, Mann–Whitney *U* test. MBs analyzed: $n = 4$ for each genotype; total fields analyzed: $n = 160$.

Supplementary Material and Methods

GFAP immunohistochemistry and quantification

MB slices were permeabilized with 0.3% TritonX-100 in PBS, incubated 16 hours at 4 °C with a mouse monoclonal antibody against GFAP (Sigma Aldrich; G6171; 1:200), then with the appropriate diluted secondary antibody (a donkey anti-mouse TRITC-conjugated; 1:200; Jackson ImmunoResearch). Negative control was obtained by omitting the primary antibody. Nuclei were stained using Hoechst 33258 (1 mg/ml; Sigma-Aldrich).

Digital pictures of the immunostained sections were collected with an Olympus FV1200 laser scanning confocal microscope. Twenty randomly representative images from four different tumors for each genotype were analyzed with the ImageJ software (National Institutes of Health, USA). The semi-quantitative analysis of the GFAP density was obtained as mean% ± SEM of the area of GFAP staining in each medulloblastoma image and was statistically analyzed by Mann-Whitney *U* test (as described in reference 1; see (2) for the detailed method). Briefly, the MB pictures were collected with identical acquisition parameters and exposure times upon setting on negative sample, to uniform

the background. Then, for each image we have set a threshold using the thresholding tool: the auto setting was selected choosing “Dark background”, which is recommended for fluorescence. Finally, we have chosen the parameter to be measured (area): for this purpose, we have also selected the “Limit to Threshold” option, which allows the measurement of the selected area only and not of the entire image.

Supplementary References

1. Maximov V, Chen Z, Wei Y, Robinson MH, Herting CJ, Shanmugam NS, et al. Tumour-associated macrophages exhibit anti-tumoural properties in Sonic Hedgehog medulloblastoma. *Nat Commun*. (2019) 10:2410. doi: 10.1038/s41467-019-10458-9.
2. Jensen EC. Quantitative analysis of histological staining and fluorescence using ImageJ. *Anat Rec (Hoboken)*. (2013) 296:378–81. doi: 10.1002/ar.22641.
